# Supplementary material for: Transcriptomic profiling of human cardiac cells predicts protein kinase inhibitor-associated cardiotoxicity
Source: Nat Commun. 2020 Sep 23;11:4809. doi: 10.1038/s41467-020-18396-7 (PMC7511315; doi:10.1038/s41467-020-18396-7)
Supplement: Supplementary file 1 — Supplementary Information [file 41467_2020_18396_MOESM1_ESM.pdf]

Supplementary Information

Transcriptomic profiling of human cardiac cells predicts protein kinase inhibitor-associated cardiotoxicity

van Hasselt et al.

Supplementary Figures 1-6

Supplementary Tables 1-3

Supplementary References

Supplementary Figures

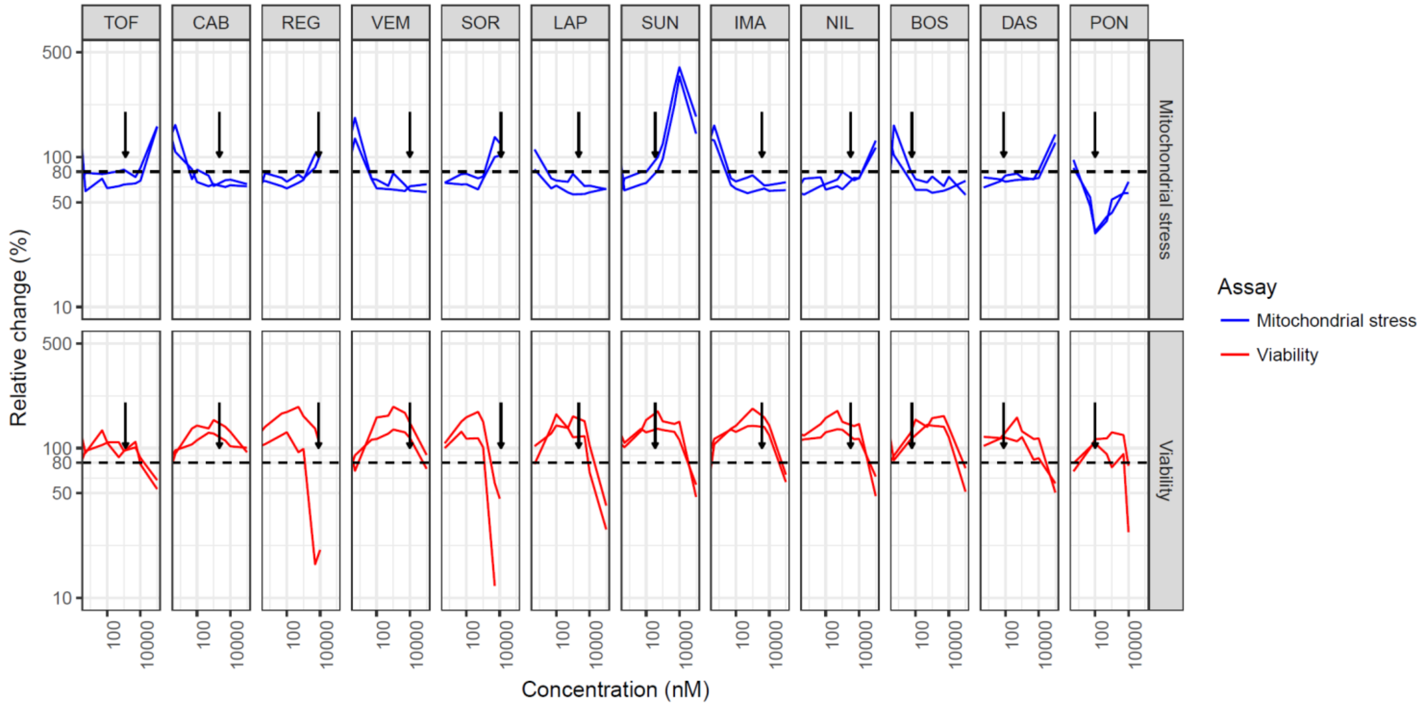

Supplementary Figure 1. Dose-response experiments for multiple selected kinase inhibitors sorted by FAERS-based clinical risk scores (left is low risk; right is high risk), measuring relative change from control in mitochondrial stress and viability. Therapeutic concentrations are indicated with vertical arrows.

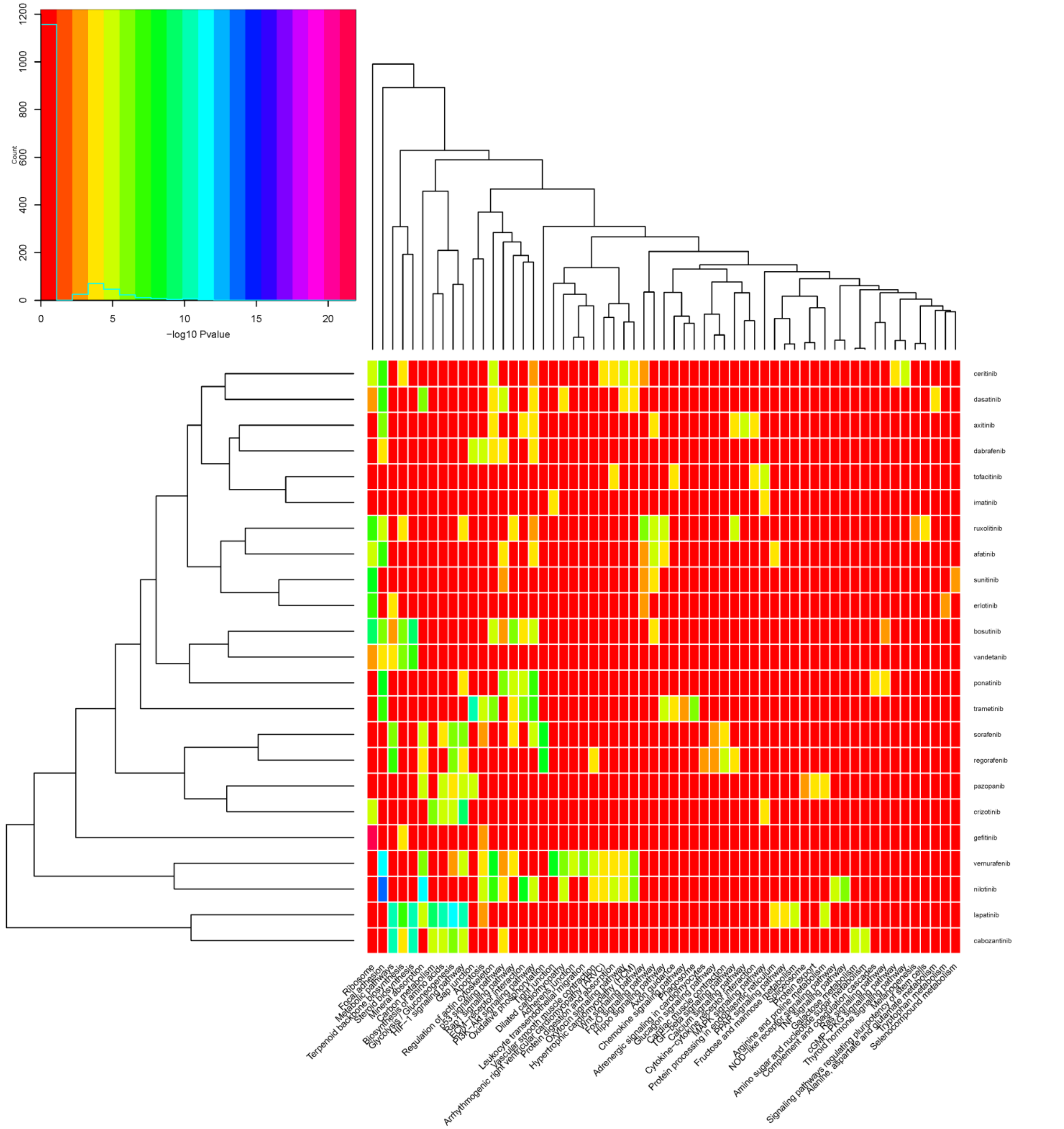

Supplementary Figure 2. Enrichment analysis of pathways in KEGG databased for each of the 23 kinase inhibitors (KIs) across 4 PromoCell cardiomyocyte cell lines based on the top 250 genes for each KI ranked by P-value.

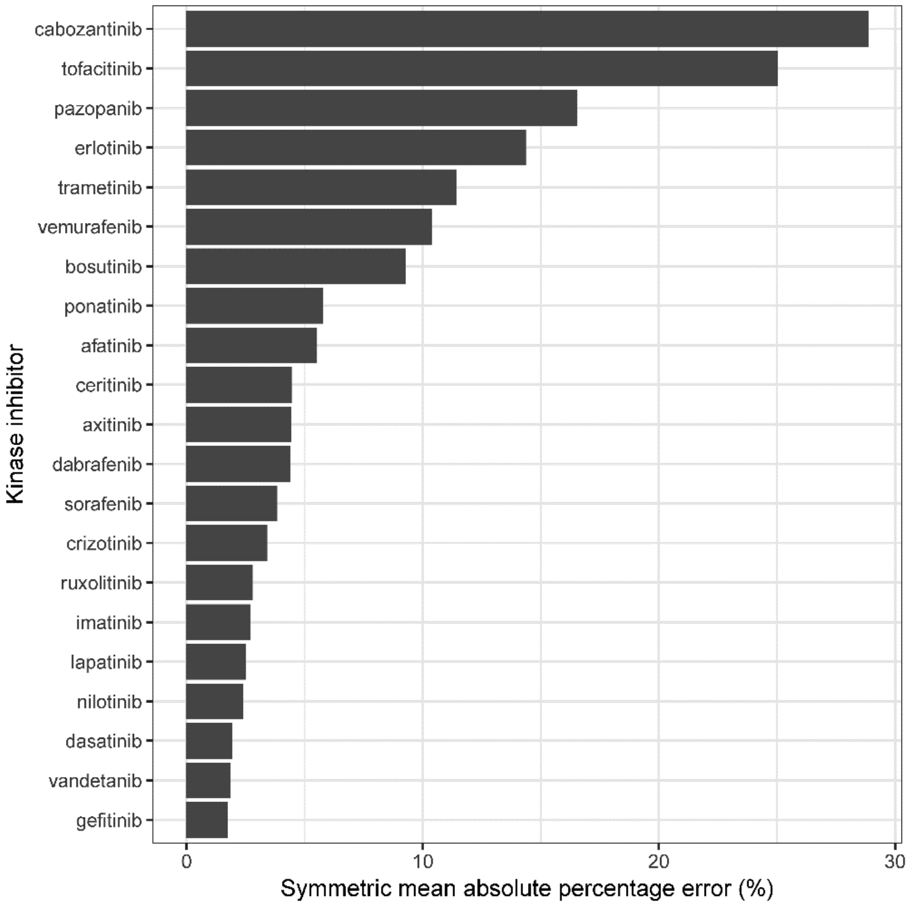

Supplementary Figure 3. Percentage of mean prediction error for left-out drugs during cross-validation. and intermediate proteins that are associated with the cardiotoxicity signature.

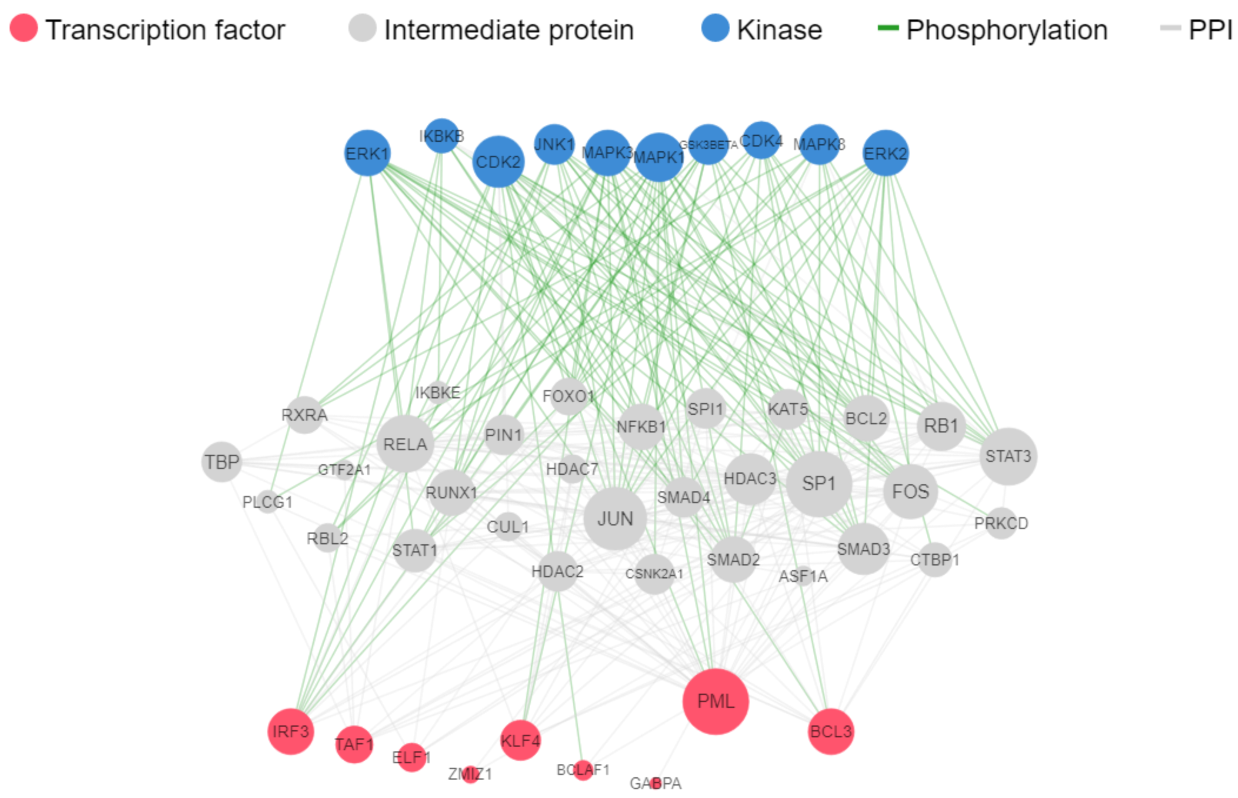

Supplementary Figure 4. Expression-to-kinase protein-protein interaction network analysis indicating key kinases, transcription factors

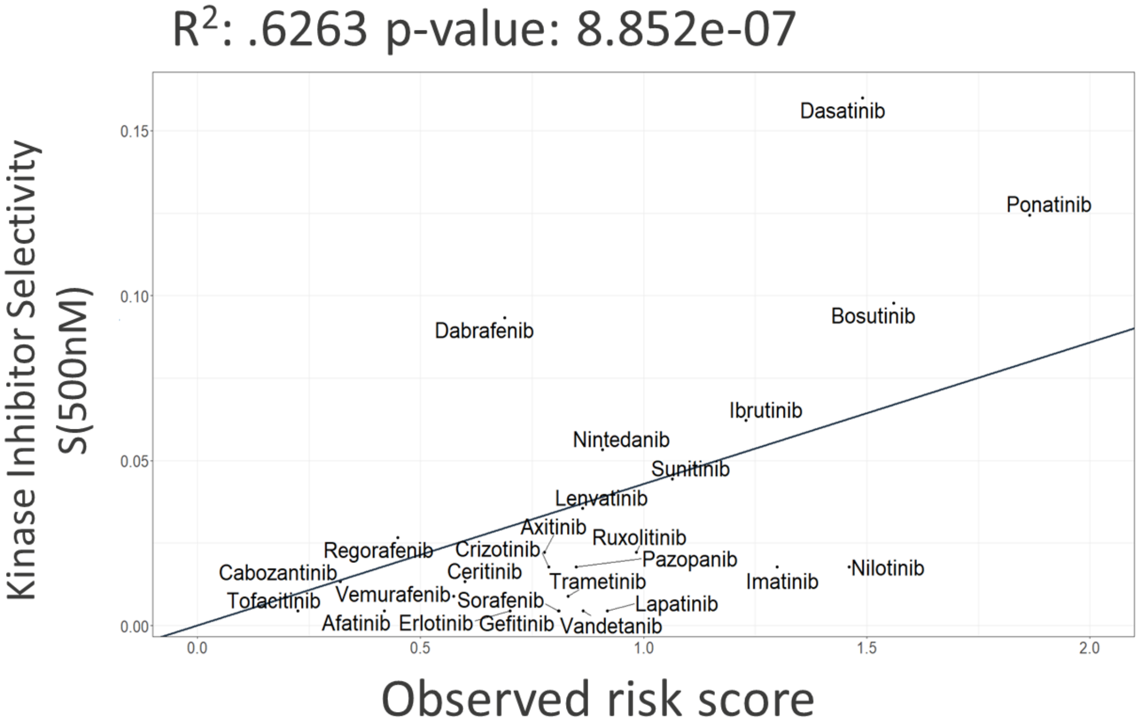

Supplementary Figure 5. Observed risk score vs kinase inhibitor selectivity at 500nM. The Wald statistic was used to test the association between kinase selectivity and observed risk score.

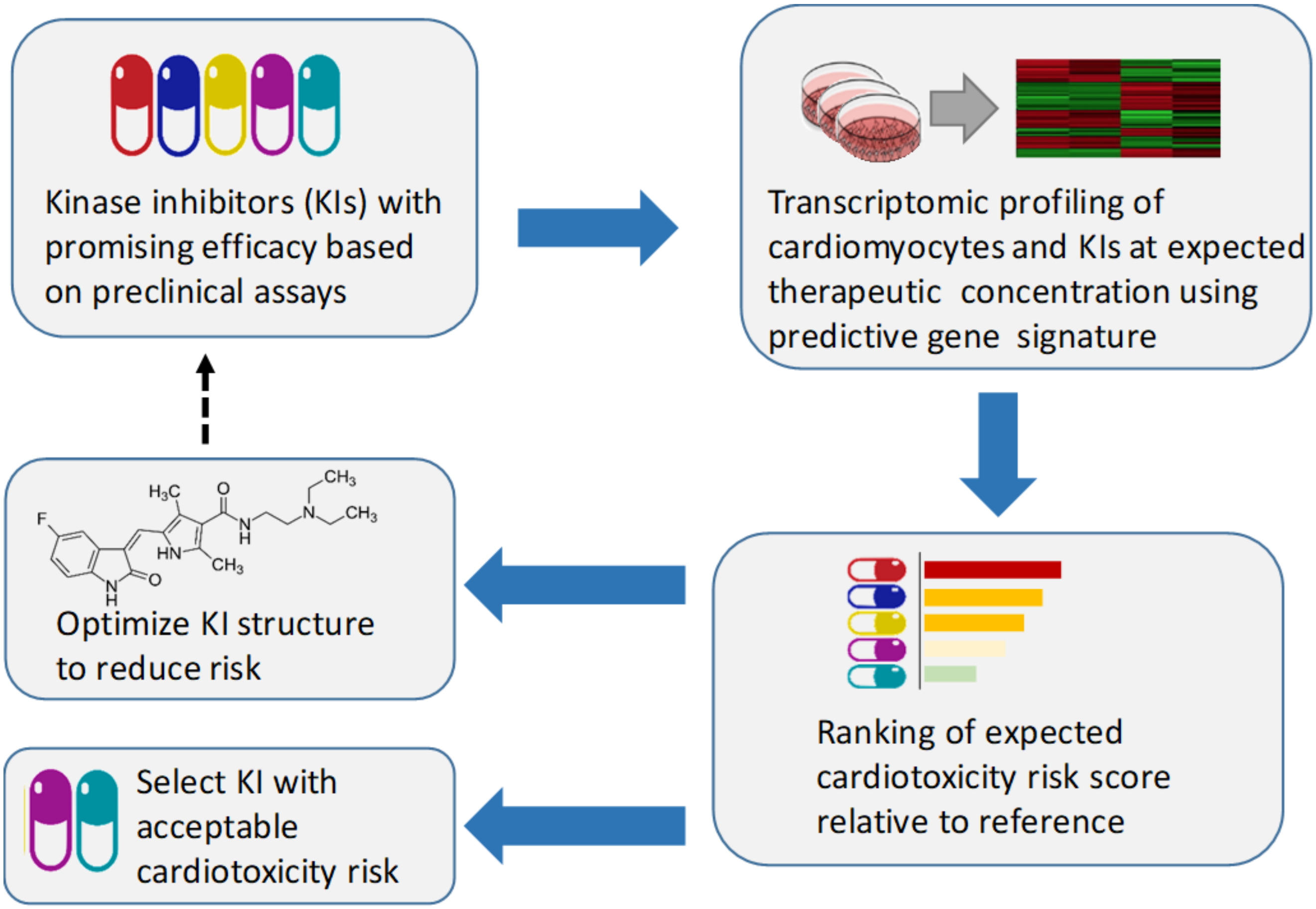

Supplementary Figure 6. Flowchart for implementation of transcriptomic cardiotoxicity signatures in preclinical drug development to reduce risk of cardiotoxicity.

Supplementary Tables

Supplementary Table 1. Typical concentrations used for experiments (exposure for 48h). Concentrations were selected based on the mean maximum drug concentrations reported in clinical studies. For dose escalation studies the highest concentration was used. All drugs were purchased from Aldrich Market Select division of Sigma, and drug purity was confirmed by HPLC-MS analysis, as described in the SOP deposited at the DToxS website.

**Drug**	**Purity**	**In Vitro (µM)**	**Reference**
Afatinib	98.80	0.05	[3334]
Axitinib	98.00	0.2	[3536]
Bosutinib	98.00	0.1	[37]
Cabozantinib	99.10	2	[38]
Ceritinib	98.85	1	[39]
Crizotinib	98.47	0.25	[40]
Dabrafenib	99.61	2.5	[4142]
Dasatinib	98.36	0.1	[4344]
Erlotinib	99.32	3	[4546]
Gefitinib	99.25	1	[47]
Imatinib	99.48	5	[48]
Lapatinib	99.00	2	[49]
Nilotinib	99.77	3	[5051]
Pazopanib	96.13	10	[52]
Ponatinib	97.29	0.1	[53]
Regorafenib	99.58	1	[54]
Ruxolitinib	99.38	1	[5556]
Sorafenib	99.73	0.5	[57]
Sunitinib	97.13	1	[5859]
Trametinib	99.24	0.1	[60]
Tofacitinib	99.85	1	[61]
Vandetanib	99.73	0.333	[62]
Vemurafenib	98.08	2	[63]

Supplementary Table 2. Overview of heart cell perturbation experiments across drugs, cell lines, plates, and numbers of replicates per experiment for which mRNAseq profiling data was included in this analysis.

**Drug**	**Nr. of cell lines**[a]	**Nr. of plates**[b]	**Number of Biological replicates/cell line**	
			Minimum	Maximum
Afatinib	4	2	3	4
Axitinib	4	1	4	4
Bosutinib	4	2	4	4
Cabozantinib	3	1	3	4
Ceritinib	4	2	3	4
Crizotinib	4	1	4	4
Dabrafenib	4	1	3	4
Dasatinib	4	2	3	4
Erlotinib	4	2	3	4
Gefitinib	4	1	4	4
Imatinib	4	1	3	4
Lapatinib	2	1	4	4
Nilotinib	4	1	3	4
Pazopanib	4	2	3	4
Ponatinib	4	2	3	4
Regorafenib	3	2	3	4
Ruxolitinib	4	2	3	4
Sorafenib	3	3	3	4
Sunitinib	4	3	3	5
Tofacitinib	4	2	3	4
Trametinib	4	2	3	4
Vandetanib	4	2	4	4
Vemurafenib	4	2	3	4

[a]Derived from different human donors.

[b]In most cases drug perturbation experiments were conducted on multiple plates that were processed in a single RNAseq-run, in order to limit the influence of potential batch-effects.

Supplementary Table 3. List of 26 genes part of the cardiotoxicity gene signature.

Coefficient	Estimate
(Intercept)	0.71042676
PRKRIP1	0.2087493
DDX3Y	-0.14451405
RPL13P5	-0.10065338
DHRS4	0.08840433
ZNF567	-0.19108461
SCO1	0.22498281
ZSCAN25	0.30633667
ZNF776	0.20654469
SNX14	-0.22973802
ZNF611	-0.06748435
MFN2	0.56373084
DSTNP2	-0.16016064
PMS2	0.18801529
NFATC2IP	-0.16092359
UBE2Q2	-0.44148014
ARHGAP12	-0.25426077
TBC1D16	-0.02704898
FAM45BP	0.33178699
GOLPH3L	-0.12777427
BCAT2	0.10332328
URM1	0.31541242
CSE1L	0.17069461
DNAJB14	0.46071526
SNORD47	-0.03746744
CASK	-0.08538788
FASTKD2	0.31397776

Supplementary References

1. Schnell D, Buschke S, Fuchs H, et al (2014) Pharmacokinetics of afatinib in subjects with mild or moderate hepatic impairment. Cancer ChemotherPharmacol 74:267–75. doi: 10.1007/s00280-014-2484-y
2. Stopfer P, Marzin K, Narjes H, et al (2012) Afatinib pharmacokinetics and metabolism after oral administration to healthy male volunteers. Cancer ChemotherPharmacol 69:1051–61. doi: 10.1007/s00280-011-1803-9
3. Pithavala YK, Chen Y, Toh M, et al (2012) Evaluation of the effect of food on the pharmacokinetics of axitinib in healthy volunteers. Cancer ChemotherPharmacol 70:103–12. doi: 10.1007/s00280-012-1888-9
4. Rugo HS, Herbst RS, Liu G, et al (2005) Phase I trial of the oral antiangiogenesis agent AG-013736 in patients with advanced solid tumors: pharmacokinetic and clinical results. J Clin Oncol 23:5474–83. doi: 10.1200/JCO.2005.04.192
5. Abbas R, Hug BA, Leister C, et al (2012) A phase I ascending single-dose study of the safety, tolerability, and pharmacokinetics of bosutinib (SKI-606) in healthy adult subjects. Cancer ChemotherPharmacol 69:221–7. doi: 10.1007/s00280-011-1688-7
6. Kurzrock R, Sherman SI, Ball DW, et al (2011) Activity of XL184 (Cabozantinib), an oral tyrosine kinase inhibitor, in patients with medullary thyroid cancer. J Clin Oncol 29:2660–6. doi: 10.1200/JCO.2010.32.4145
7. Shaw AT, Kim D-W, Mehra R, et al (2014) Ceritinib in ALK-rearranged non-small-cell lung cancer. N Engl J Med 370:1189–97. doi: 10.1056/NEJMoa1311107
8. Kwak E, Camidge D, Clark J, et al (2009) Clinical activity observed in a phase I dose escalation trial of an oral c-MET and ALK inhibitor. J Clin Oncol 27:
9. Ouellet D, Gibiansky E, Leonowens C, et al (2014) Population pharmacokinetics of dabrafenib, a BRAF inhibitor: effect of dose, time, covariates, and relationship with its metabolites. J ClinPharmacol 54:696–706. doi: 10.1002/jcph.263
10. Falchook GS, Long G V, Kurzrock R, et al (2012) Dabrafenib in patients with melanoma, untreated brain metastases, and other solid tumours: a phase 1 dose-escalation trial. Lancet (London, England) 379:1893–901. doi: 10.1016/S0140-6736(12)60398-5
11. Dai CL, Tiwari AK, Wu CP, et al (2008) Lapatinib (Tykerb, GW572016) reverses multidrug resistance in cancer cells by inhibiting the activity of ATP-binding cassette subfamily B member 1 and G member 2. Cancer Res 68:7905–7914. doi: 10.1158/0008-5472.CAN-08-0499
12. McCaig AM, Cosimo E, Leach MT, Michie AM (2012) Dasatinib inhibits CXCR4 signaling in chronic lymphocytic leukaemia cells and impairs migration towards CXCL12. PLoS One 7:e48929. doi: 10.1371/journal.pone.0048929
13. Herbst RS, Prager D, Hermann R, et al (2005) TRIBUTE: A phase III trial of erlotinib hydrochloride (OSI-774) combined with carboplatin and paclitaxel chemotherapy in advanced non-small-cell lung cancer. J Clin Oncol 23:5892–5899. doi: 10.1200/JCO.2005.02.840
14. Soulieres D, Senzer NN, Vokes EE, et al (2004) Multicenter phase II study of erlotinib, an oral epidermal growth factor receptor tyrosine kinase inhibitor, in patients with recurrent or metastatic squamous cell cancer of the head and neck. J Clin Oncol 22:77–85. doi: 10.1200/JCO.2004.06.075
15. Hirano S, Sano K, Takeda Y, et al (2012) The pharmacokinetics and long-term therapeutic effects of gefitinib in patients with lung adenocarcinoma harboring the epidermal growth factor receptor(EGFR)mutation. Gan To Kagaku Ryoho 39:1501–6.
16. Demetri GD, Wang Y, Wehrle E, et al (2009) Imatinib plasma levels are correlated with clinical benefit in patients with unresectable/metastatic gastrointestinal stromal tumors. J Clin Oncol 27:3141–3147. doi: 10.1200/JCO.2008.20.4818
17. LoRusso PM, Jones SF, Koch KM, et al (2008) Phase I and pharmacokinetic study of lapatinib and docetaxel in patients with advanced cancer. J Clin Oncol 26:3051–6. doi: 10.1200/JCO.2007.14.9633

18. Trent J, Molimard M (2011) Pharmacokinetics and pharmacodynamics of nilotinib in gastrointestinal stromal tumors. Semin Oncol 38 Suppl 1:S28–33. doi: 10.1053/j.seminoncol.2011.01.014
19. Tanaka C, Yin OQP, Sethuraman V, et al (2010) Clinical pharmacokinetics of the BCR-ABL tyrosine kinase inhibitor nilotinib. ClinPharmacolTher 87:197–203. doi: 10.1038/clpt.2009.208
20. Hurwitz HI, Dowlati A, Saini S, et al (2009) Phase I Trial of Pazopanib in Patients with Advanced Cancer rial of Pazopanib in Patients with Advanced Cancer. 4220–4227. doi: 10.1158/1078-0432.CCR-08-2740
21. Narasimhan NI, Dorer DJ, Niland K, et al (2013) Effects of ketoconazole on the pharmacokinetics of ponatinib in healthy subjects. J ClinPharmacol 53:974–81. doi: 10.1002/jcph.109
22. Rey J-B, Launay-Vacher V, Tournigand C (2015) Regorafenib as a single-agent in the treatment of patients with gastrointestinal tumors: an overview for pharmacists. Target Oncol 10:199–213. doi: 10.1007/s11523-014-0333-x
23. Shi JG, Chen X, McGee RF, et al (2011) The pharmacokinetics, pharmacodynamics, and safety of orally dosed INCB018424 phosphate in healthy volunteers. J ClinPharmacol 51:1644–54. doi: 10.1177/0091270010389469
24. Chen X, Shi JG, Emm T, et al (2014) Pharmacokinetics and pharmacodynamics of orally administered ruxolitinib (INCB018424 phosphate) in renal and hepatic impairment patients. ClinPharmacol drug Dev 3:34–42. doi: 10.1002/cpdd.77
25. Abou-Alfa GK, Schwartz L, Ricci S, et al (2006) Phase II study of sorafenib in patients with advanced hepatocellular carcinoma. J Clin Oncol 24:4293–300. doi: 10.1200/JCO.2005.01.3441
26. Minkin P, Zhao M, Chen Z, et al (2008) Quantification of sunitinib in human plasma by high-performance liquid chromatography-tandem mass spectrometry. J Chromatogr B Anal Technol Biomed Life Sci 874:84–88. doi: 10.1016/j.jchromb.2008.09.007
27. Faivre S, Delbaldo C, Vera K, et al (2006) Safety, pharmacokinetic, and antitumor activity of SU11248, a novel oral multitarget tyrosine kinase inhibitor, in patients with cancer. J Clin Oncol 24:25–35. doi: 10.1200/JCO.2005.02.2194
28. Kim KB, Kefford R, Pavlick AC, et al (2013) Phase II study of the MEK1/MEK2 inhibitor Trametinib in patients with metastatic BRAF-mutant cutaneous melanoma previously treated with or without a BRAF inhibitor. J Clin Oncol 31:482–9. doi: 10.1200/JCO.2012.43.5966
29. Dowty ME, Lin J, Ryder TF, et al (2014) The pharmacokinetics, metabolism, and clearance mechanisms of tofacitinib, a janus kinase inhibitor, in humans. Drug MetabDispos 42:759–73. doi: 10.1124/dmd.113.054940
30. Martin P, Oliver S, Robertson J, et al (2011) Pharmacokinetic drug interactions with vandetanib during coadministration with rifampicin or itraconazole. Drugs R D 11:37–51. doi: 10.2165/11586980-000000000-00000
31. Grippo JF, Zhang W, Heinzmann D, et al (2014) A phase I, randomized, open-label study of the multiple-dose pharmacokinetics of vemurafenib in patients with BRAF V600E mutation-positive metastatic melanoma. Cancer ChemotherPharmacol 73:103–11. doi: 10.1007/s00280-013-2324-5
32 DtoxS web site: www.dtoxs.org
