# Supplementary material for: Transcriptomic profiling of human cardiac cells predicts protein kinase inhibitor-associated cardiotoxicity
Source: Nat Commun. 2020 Sep 23;11:4809. doi: 10.1038/s41467-020-18396-7 (PMC7511315; doi:10.1038/s41467-020-18396-7)
Supplement: Supplementary file 3 — Reporting Summary [file 41467_2020_18396_MOESM3_ESM.pdf]

nature research

Corresponding author(s): Ravi Iyengar, Evren Azeloglu, Avner Schlessinger

Last updated by author(s): Jul 9, 2020

Reporting Summary

Nature Research wishes to improve the reproducibility of the work that we publish. This form provides structure for consistency and transparency in reporting. For further information on Nature Research policies, see our Editorial Policies and the Editorial Policy Checklist.

Statistics

For all statistical analyses, confirm that the following items are present in the figure legend, table legend, main text, or Methods section.

n/a	Confirmed	
☐	☒	The exact sample size (*n*) for each experimental group/condition, given as a discrete number and unit of measurement
☐	☒	A statement on whether measurements were taken from distinct samples or whether the same sample was measured repeatedly
☐	☒	The statistical test(s) used AND whether they are one- or two-sided<br>*Only common tests should be described solely by name; describe more complex techniques in the Methods section.*
☐	☒	A description of all covariates tested
☐	☒	A description of any assumptions or corrections, such as tests of normality and adjustment for multiple comparisons
☐	☒	A full description of the statistical parameters including central tendency (e.g. means) or other basic estimates (e.g. regression coefficient) AND variation (e.g. standard deviation) or associated estimates of uncertainty (e.g. confidence intervals)
☐	☒	For null hypothesis testing, the test statistic (e.g. *F*, *t*, *r*) with confidence intervals, effect sizes, degrees of freedom and *P* value noted<br>*Give P values as exact values whenever suitable.*
☒	☐	For Bayesian analysis, information on the choice of priors and Markov chain Monte Carlo settings
☐	☒	For hierarchical and complex designs, identification of the appropriate level for tests and full reporting of outcomes
☒	☐	Estimates of effect sizes (e.g. Cohen's *d*, Pearson's *r*), indicating how they were calculated

Our web collection on statistics for biologists contains articles on many of the points above.

Software and code

Policy information about availability of computer code

Data collection	Adverse event (ADR) frequencies from the FDA Adverse Event Reporting System (FAERS) were obtained from the AERSmine resource V2017-07-11
Data analysis	ombined standard RNA-seq files were aligned to the reference human genome hg38 using the STAR software suite V 2.7<br>The gene read-count tables were then subjected to differential gene expression analysis using the R package EdgeR V 3.10<br>The FAERS-derived risk RORs for CT were regressed against the KI associated vectors of mean fold change values across the four cell lines using an elastic net regression model: R version 3.4.3, package glmnet, version 2.0-16<br>Enrichment analysis was performed based on a one-tailed Fisher's exact test using R (package stats)<br>Protein-protein interaction network (PPI) analysis of genes associated with cardiotoxicity was conducted using Expression 2 Kinases (2018)<br>RDkit (www.rdkit.org) was used to generate chemical fingerprints and compute pairwise Tanimoto coefficients (Tc) between the 26 tested kinase inhibitors.<br>For each pair of inhibitors, we first calculated the Tc using four chemical fingerprints found within the RDKit package: Morgan_2 2,048-bit (ECFP4), Morgan_1 2,048-bit (ECFP2) , Daylightlike, and MACCS.<br>Chemical structures were drawn using Marvin (www.chemaxon.com) based on SMILES strings obtained from PubChem.<br>Custom code and scripts were used to generate figures and are available at https://github.com/dtoxs/FAERS-Analysis

For manuscripts utilizing custom algorithms or software that are central to the research but not yet described in published literature, software must be made available to editors and reviewers. We strongly encourage code deposition in a community repository (e.g. GitHub). See the Nature Research guidelines for submitting code & software for further information.

Data

Policy information about availability of data

All manuscripts must include a data availability statement. This statement should provide the following information, where applicable:

- Accession codes, unique identifiers, or web links for publicly available datasets
- A list of figures that have associated raw data
- A description of any restrictions on data availability

Adverse Drug Reactionss in the FAERS database (https://www.fda.gov/drugs/questions-and-answers-fdas-adverse-event-reporting-system-faers/fda-adverse-event-reporting-system-faers-latest-quarterly-data-files) are organized according to MedDRA V 20.0 (www.meddra.org)
For enrichment of pathways and biological processes we used the KEGG database (2016) (https://www.genome.jp/kegg/)
For enrichment of protein kinases we used the KEA database (2015) (https://www.maayanlab.net/KEA2/)
Kinome-wide kinase inhibitor-binding (Kd) profiling data was obtained from Klaegar et al. 2017 (DOI: 10.1126/science.aan4368)
All processed RNAseq data and the curated version-controlled standard operating procedures featured in this study can be downloaded freely at (https://martip03.u.hpc.mssm.edu/data.php)
Tissue expression data was obtained from GTEx (V8) (https://www.gtexportal.org/home/datasets)
or the LINCS Data Portal (http://dev3.ccs.miami.edu:8080/signatures/datasets/LDG-1444/).
Raw transcriptomics data can be accessed through the Gene Expression Omnibus (GEO) repository with accession numbers GSE146096 and GSE146097.

Field-specific reporting

Please select the one below that is the best fit for your research. If you are not sure, read the appropriate sections before making your selection.

☒ Life sciences ☐ Behavioural & social sciences ☐ Ecological, evolutionary & environmental sciences

For a reference copy of the document with all sections, see nature.com/documents/nr-reporting-summary-flat.pdf

Life sciences study design

All studies must disclose on these points even when the disclosure is negative.

Sample size	Sample size was not predetermined. We obtained four independent cardiomyocyte cell lines from PromoCell, each derived from a different human subject. We selected 23 different kinase inhibitors approved by the FDA at the time of study design. Each drug was tested in four replicates for each of the four cell lines. We did not perform power analysis a priori; we chose samples sizes based on prior experience with similar transcriptomic experiments. We ran multiple hypotheses corrected post hoc analyses to ensure adequate statistical power.
Data exclusions	No data was excluded
Replication	For each drug treatment on a given cell line, there were four replicate treatments that were independently subjected to transcriptomic profiling. These were not technical replicates. They were all obtained from independently cultured cell line treated at a different time point. In most cases, data from all four replicates were used to identify ranked list of genes. Details are provided at the LINCS DCIC Data Portal, where all of the raw and processed data is freely available.
Randomization	These are cell-based experiments, where cell density, drug concentration and time of treatment and culture were precisely controlled. Similarly, library preparation and sequencing were performed for all replicates in 6 different batches, whereby presence of potential batch effects were independently screened as part of quality control. Hence other than procedures described under materials and methods, no additional procedures were used to control for covariates.
Blinding	The investigators were not blinded. Our study did not involve any experimental group allocation.

Reporting for specific materials, systems and methods

We require information from authors about some types of materials, experimental systems and methods used in many studies. Here, indicate whether each material, system or method listed is relevant to your study. If you are not sure if a list item applies to your research, read the appropriate section before selecting a response.

Materials & experimental systems

n/a	Involved in the study
[x]	[ ] Antibodies
[ ]	[x] Eukaryotic cell lines
[x]	[ ] Palaeontology and archaeology
[x]	[ ] Animals and other organisms
[x]	[ ] Human research participants
[x]	[ ] Clinical data
[x]	[ ] Dual use research of concern

Methods

n/a	Involved in the study
[x]	[ ] ChIP-seq
[x]	[ ] Flow cytometry
[x]	[ ] MRI-based neuroimaging

Eukaryotic cell lines

Policy information about cell lines

Cell line source(s)	Cells were purchased from PromoCell GmbH.
Authentication	We performed RT-PCR and immunofluorescence for cell type authentication. Details are provided on DToxS website.
Mycoplasma contamination	We used a PCR-based mycoplasma test to validate no Mycoplasma contamination.
Commonly misidentified lines (See ICLAC register)	None were used in this study.
